# Genome-Wide Identification of the *LsaPHR1* Gene Family and Preliminary Functional Validation of *LsaPHR1.1* in Phosphorus Tolerance in *Lactuca sativa*

**DOI:** 10.3390/ijms262110466

**Published:** 2025-10-28

**Authors:** Yuxuan Qian, Xue Liu, Baoju Wang, Dayong Li, Zhanhui Wu, Jing Tong

**Affiliations:** 1 Key Laboratory of Urban Agriculture (North) of Ministry of Agriculture, Beijing Vegetable Research Center, Beijing Academy of Agriculture and Forestry Science, Beijing 100097, China; 13718663432@163.com (Y.Q.); liuxue1979@126.com (X.L.); wangbaoju@nercv.org (B.W.); lidayong@nercv.org (D.L.); 2 State Key Laboratory of Vegetable Biobreeding, Beijing Vegetable Research Center, Beijing Academy of Agriculture and Forestry Science, Beijing 100097, China; 3 National Engineering Research Center for Vegetables, Beijing Vegetable Research Center, Beijing Academy of Agriculture and Forestry Science, Beijing 100097, China

**Keywords:** PHR1, phosphorus, lettuce, stress, transgenic

## Abstract

Phosphorus (P) is a limiting nutrient for plant growth and productivity. Improving P use efficiency is important for crop production. In *Lactuca sativa* (lettuce), five phosphate starvation response 1 (*PHR1*) genes were identified and characterized through a bioinformatics approach. The expression patterns of *LsaPHR1*s were examined using qRT-PCR under various treatments, including devoid phosphorus (DP), low phosphorus (LP), high phosphorus (HP), darkness, ABA, IAA, and MeJA. The results indicate that *LsaPHR1*s in lettuce responded to phosphorus stress, hormones, and darkness. Furthermore, we engineered *LsaPHR1.1* knock-out mutants via CRISPR/Cas9-mediated genome editing. Then, the mutants were subjected to phosphorus stress (DP, LP, and HP). In contrast to WT, the mutants improved nitrate and ammonium contents, increased antioxidant enzyme activity, and elevated antioxidant and chlorophyll contents. Our results offer a potential strategy for improving phosphorus stress tolerance in lettuce, which holds great significance for maintaining yield and quality.

## 1. Introduction

Nitrogen (N), phosphorus (P), and potassium (K) are critical macronutrients required for optimal plant growth, development, and yield [1,2]. Among these, P plays a particularly crucial role in supporting plant physiological processes and enhancing productivity [3]. However, in both acidic and alkaline soil conditions, P tends to form insoluble precipitates by binding with cations like iron (Fe), aluminum (Al), and calcium (Ca), significantly reducing its bioavailability for plant uptake [4]. To overcome this limitation, farmers often apply large amounts of phosphate fertilizers, a practice that not only increases production costs but also leads to environmental degradation due to nutrient runoff. Given these challenges, elucidating the genetic and biochemical pathways that enhance P use efficiency in plants is essential for promoting sustainable agriculture, especially in regions with low soil P availability. Advancements in this field could pave the way for improved crop varieties or precision fertilization techniques, reducing dependence on excessive fertilizer inputs.

To survive in phosphorus-deficient environments, plants have developed an elaborate gene regulatory network dedicated to phosphate (Pi) uptake and homeostasis. Key components of this system include phosphate starvation response 1 (PHR1), phosphate starvation-induced 1 (IPS1), phosphate 2 (PHO2), and phosphate transporters (PTs), which collectively mediate Pi acquisition and distribution [5,6]. PHR1, a central transcription factor in Pi signaling, orchestrates the plant’s response to Pi deficiency by modulating downstream gene expression [7]. Genome-wide studies have identified PHR1 homologs in various species, including model and woody plants. For instance, in *Arabidopsis thaliana*, disruption of AtPHR1 alters lipid metabolism, primary/secondary metabolic pathways, and photosynthetic efficiency, affecting root development and anthocyanin biosynthesis [8]. Similarly, functional studies in apple (*Malus domestica*) and poplar (*Populus* spp.) have confirmed the role of PHR1 in low-Pi tolerance [9,10]. Under Pi-deficient conditions, cellular Pi and ATP levels decline, triggering PPIP5K-dependent metabolic shifts that favor the conversion of inositol polyphosphates (InsPs) from InsP7 to InsP6 rather than InsP8 [11]. Since InsP8 stabilizes the SPX-PHR complex, its reduction under low Pi weakens this interaction, liberating PHR from its SPX repressors [12]. The freed PHR translocates into the nucleus, activating phosphate starvation-inducible (PSI) genes to enhance Pi scavenging [13]. Conversely, under Pi-sufficient conditions, PPIP5K promotes InsP8 synthesis, reinforcing the SPX-PHR interaction and suppressing PHR activity. This inhibition blocks low-Pi signaling, ensuring metabolic homeostasis when Pi is abundant [12,13].

As a popular member of the Asteraceae family, lettuce (*Lactuca sativa* L.) is favored in culinary applications due to its appealing taste, distinctive fragrance, and satisfying crunchy texture [14]. However, the identification of the lettuce *PHR1* (*LsaPHR1*) gene family has not yet been reported. Using bioinformatic analysis, we conducted a comprehensive identification and characterization of the *PHR1* gene family in lettuce. The expression patterns of *LsaPHR1*s were examined under devoid phosphorus, low phosphorus, high phosphorus, darkness, ABA, IAA, and MeJA treatments using qRT-PCR. Additionally, we generated transgenic lettuce by editing *LsaPHR1.1* to validate its function in phosphorus (DP, LP, and HP) tolerance, with the aim of improving the efficiency of phosphorus utilization and increasing lettuce yield.

## 2. Results

### 2.1. Identification of the PHR1 Genes in the Lettuce Genome

The lettuce genome was found to contain five PHR1 homologs based on BlastP identification. The LsaPHR1 proteins exhibited varying lengths from 435 amino acids (LsaPHR1.1) to 261 amino acids (LsaPHR1.5), with molecular weights ranging between 48,138.67 and 28,849.99 Da. The calculated isoelectric points (pI) vary from 5.30 (LsaPHR1.3) to 6.60 (LsaPHR1.5), indicating that all of the LsaPHR1s are acidic proteins (pI < 7) in nature. All identified LsaPHR1s display negative GRAVY values, demonstrating their hydrophilic characteristics. Subcellular localization predictions revealed that five LsaPHR1s are localized to the nucleus (Table 1).

### 2.2. Phylogenetic Analysis of the LsaPHR1 Gene Family

**Figure 1:**
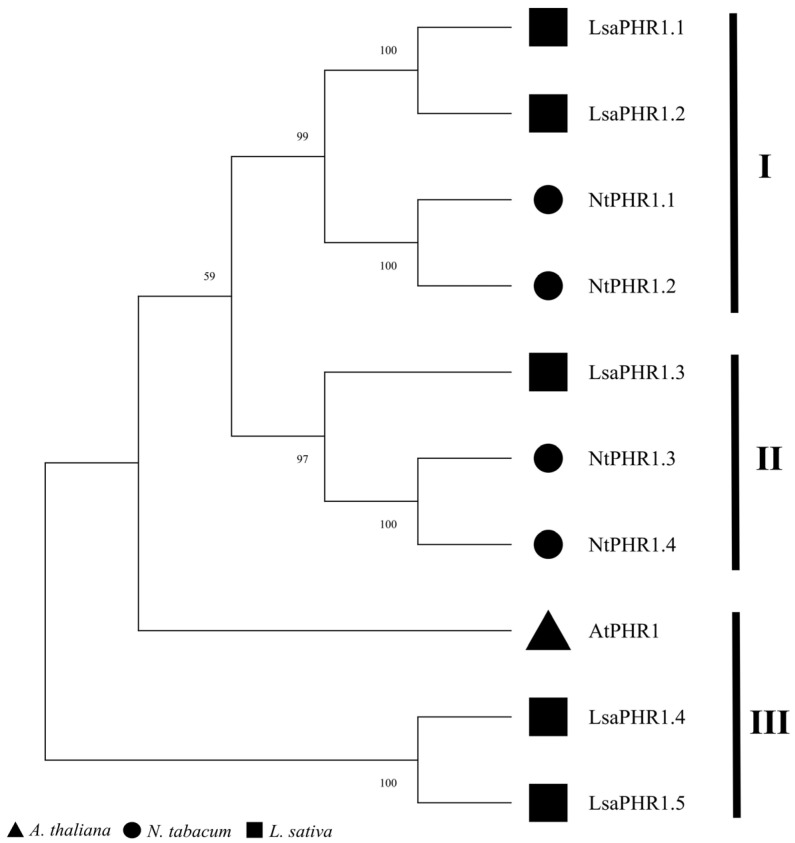
Phylogenetic relationships among PHR1s.

A phylogenetic analysis was performed using MEGA 11 to assess the evolutionary conservation of LsaPHR1 proteins relative to their orthologs in other model plant species. Based on homology and classification, PHR1s were grouped into three subclades, comprising four and three (clade II, III) PHR1 proteins. The clustering pattern within subclades indicates strong phylogenetic conservation, implying possible gene family contraction or duplication events throughout evolution (Figure 1).

### 2.3. Conservation and Structural Analysis of LsaPHR1s

**Figure 2:**
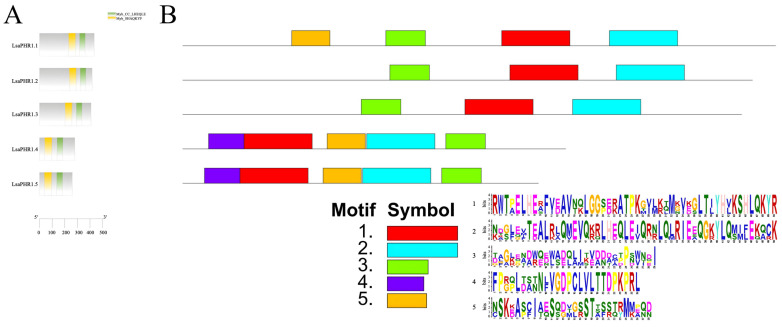
Functional domains and conserved motifs of LsaPHR1s. (**A**) Functional domains of LsaPHR1s. (**B**) Conserved motifs of LsaPHR1s.

**Figure 3:**
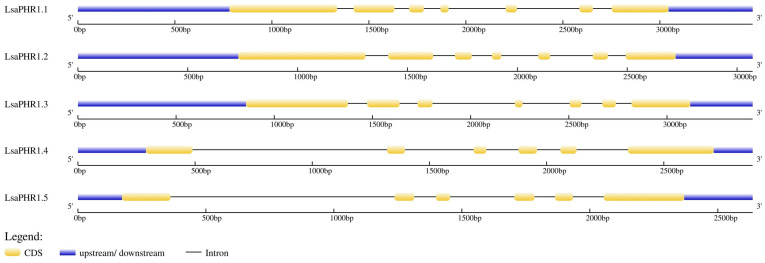
Structures of *LsaPHR1* genes.

Functional characterization of LsaPHR1 proteins was performed through domain architecture analysis using CDD and motif identification via MEME, providing insights into their structural features. Notably, all LsaPHR1s have Myb_CC_LHEQLE domains (motif 2, NDGLEVTEALRLQMEVQKRLHEQLEIQRNLQLRIEEQGKYLQMIFEKQCK) and Myb_SHAQKYF domains (motif 1, RWTPELHEAFVEAVNQLGGSERATPKGVLKQMGVEGLTIYHVKSHLQKYR). However, an additional motif (motif 3, DAGKEADWODWADOLITVDDACPPNWNDI) is also ubiquitous in all LsaPHR1 proteins. Interestingly, motif 5 (NSKEASCIAESODVGSSTSSSTRMMPOD) is absent in LsaPHR1.2 and LsaPHR1.3, but motif 4 (FPGPLDANNFVGDPCLVLTTDPKPRL) is only present in LsaPHR1.4 and LsaPHR1.5 (Figure 2). As shown in Figure 3, the gene structure analysis revealed that *LsaPHR1.1*, *LsaPHR1.2*, and *LsaPHR1.3* have seven exons each, while *LsaPHR1.4* and *LsaPHR1.5* contain six exons each. Overall, the results suggest that LsaPHR1.4 and LsaPHR1.5 differ from the other LsaPHR1s in terms of conserved motifs and gene structure.

### 2.4. Chromosomal Distribution and Cis-Acting Elements of LsaPHR1s

**Figure 4:**
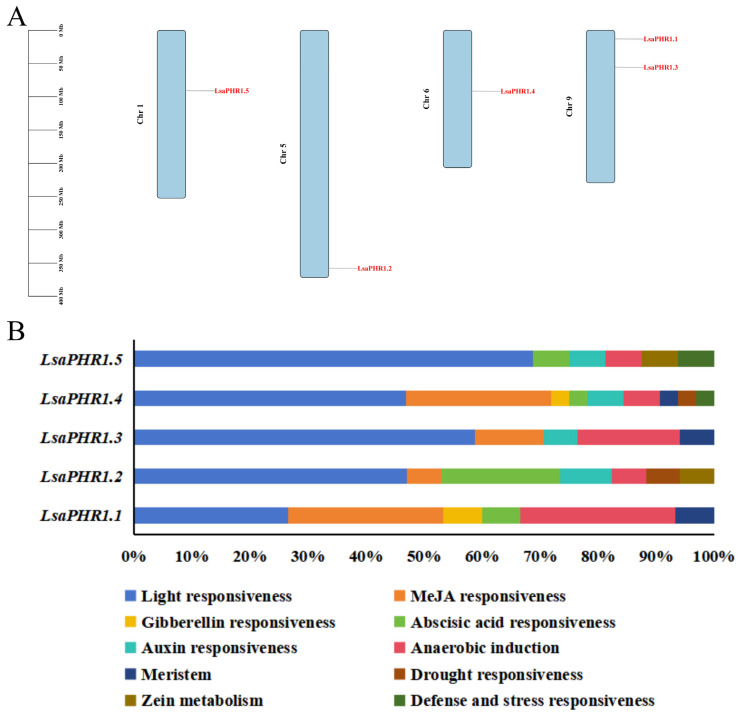
Chromosomal localization and cis-acting elements of *LsaPHR1*s. (**A**) Distribution of *LsaPHR1*s across lettuce chromosomes. (**B**) Percentage of each cis-acting element in the promoter region of *LsaPHR1*s.

The five *LsaPHR1* genes exhibit an uneven chromosomal distribution among the four lettuce chromosomes. Chromosome 9 has two *LsaPHR1*s, and chromosomes 1, 5, and 6 have one *LsaPHR1* each, whereas the other chromosomes are devoid of any members of this gene family (Figure 4A). Bioinformatic analysis of the 2 kb upstream promoter sequences of *LsaPHR1* genes was conducted using PlantCARE to characterize cis-acting elements involved in developmental processes and stress adaptation. The investigation revealed an abundance of photoresponsive motifs, highlighting potential light-mediated transcriptional regulation. Additionally, multiple phytohormone-related elements were detected, encompassing response motifs for auxins, gibberellins, jasmonates, and ABA, indicating the intricate hormonal modulation of gene expression. Moreover, elements associated with anaerobic induction were prevalent. These results indicate that *LsaPHR1*s respond to various hormones or environmental signals (Figure 4B).

### 2.5. Expression Patterns of LsaPHR1s Under Phosphate, Phytohormone, and Dark Treatments

**Figure 5:**
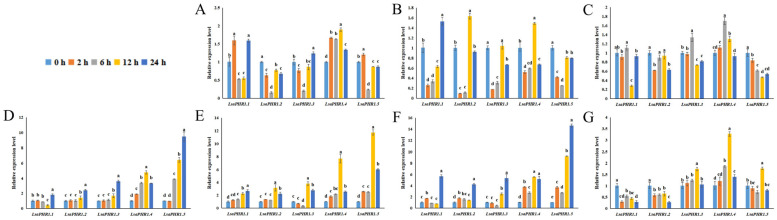
qRT-PCR analysis of *LsaPHR1*s under (**A**) DP, (**B**) LP, (**C**) HP, (**D**) ABA, (**E**) IAA, (**F**) MeJA, and (**G**) dark treatments. The relative expression levels were calculated using the 2^−∆∆Ct^ method with three replicates; *18S rRNA* was used as the reference gene. Vertical bars indicate standard deviations, and different lowercase letters above the bars indicate significant differences (0.01 < *p* < 0.05).

The study evaluated how *LsaPHR1* genes respond transcriptionally to abiotic stresses, including variations in phosphorus, hormone signaling, and the absence of light (Figure 5). In the early stages, *LsaPHR1.1*, *LsaPHR1.2*, *LsaPHR1.3*, and *LsaPHR1.5* responded to DP rapidly, suggesting its sustained role in DP tolerance. Under LP treatment, *LsaPHR1*s were initially downregulated rapidly. In HP stress, *LsaPHR1.2* exhibited rapid downregulation at the beginning. Under MeJA treatment, *LsaPHR1.1*, *LsaPHR1.2*, *LsaPHR1.4*, and *LsaPHR1.5* exhibited upregulation, followed by downregulation at 6 h.

Following ABA treatment, *LsaPHR1.1*, *LsaPHR1.2*, *LsaPHR1.3*, and *LsaPHR1.5* did not change significantly in the early stages but then gradually increased (only *LsaPHR1.1* decreased at 12 h), peaking at 24 h. During IAA treatment, *LsaPHR1.2*, *LsaPHR1.3*, *LsaPHR1.4*, and *LsaPHR1.5* exhibited rapid upregulation at 12 h. Under MeJA treatment, all *LsaPHR1*s were upregulated rapidly at 12 or 24 h.

Some *LsaPHR1* genes remained upregulated or downregulated for a long period of time, or exhibited a slow and persistent process of change. For example, during DP and dark treatments, *LsaPHR1.4* was initially upregulated, peaking at 12 h, followed by a decrease. During IAA treatment, *LsaPHR1.1* was upregulated gradually. Furthermore, prolonged dark treatment led to marked downregulation of both *LsaPHR1.1* and *LsaPHR1.2* transcript levels.

On the whole, the expression patterns of *LsaPHR1*s under different treatments are complex. These collective findings suggest that *LsaPHR1* genes function as integrators of phosphorus signaling, hormone perception, and dark stress responses in *Lactuca sativa*.

### 2.6. Editing LsaPHR1.1 Has Little Effect on Lettuce Morphology and Phosphorus Content

**Figure 6:**
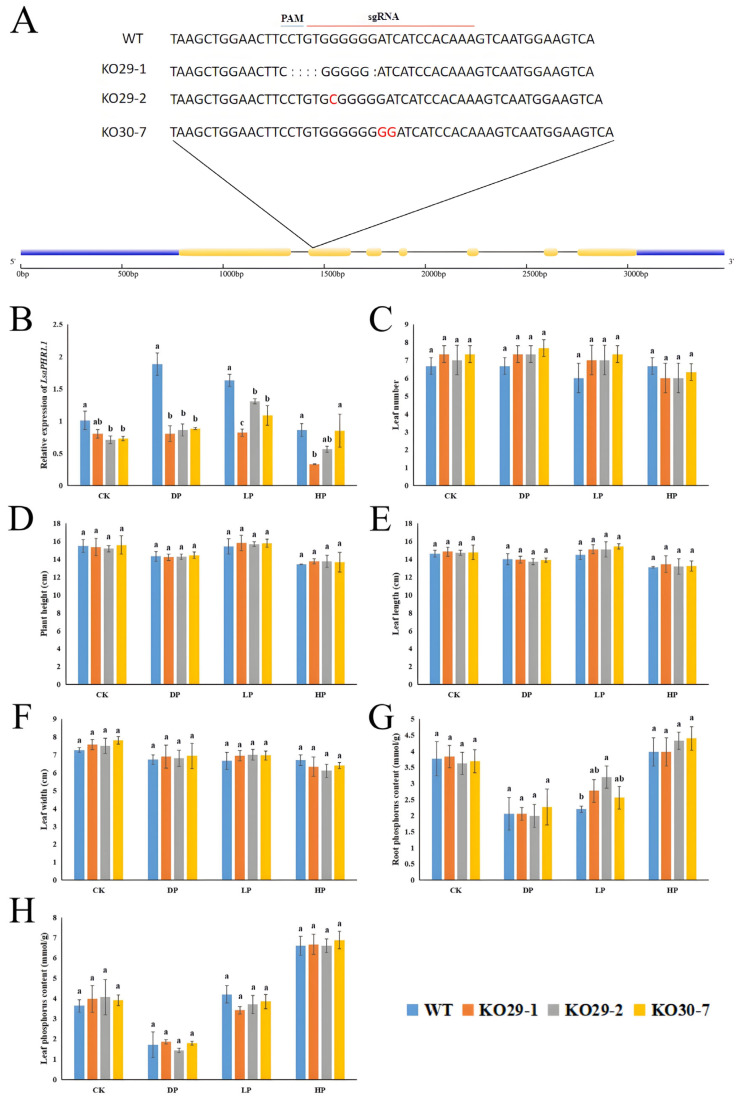
Analysis of key agronomic traits of wild-type and transgenic lettuce under phosphorus stress. (**A**) Schematic diagram of the gene editing *LsaPHR1.1* and modified sequences of *LsaPHR1.1* in the three knockout mutants. The guide RNA (sgRNA) and the sequence recognized by the Cas9 nuclease (PAM sequences) were overlined in red and blue, respectively. Blue represents exons, yellow represents CDS. (**B**) Relative expression levels of *LsaPHR1.1*. (**C**) Leaf number. (**D**) Plant height. (**E**) Leaf length. (**F**) Leaf width. (**G**) Root phosphorus content. (**H**) Leaf phosphorus content. Error bars represent the standard deviations of three replicates, and different lowercase letters above the bars indicate significant differences (0.01 < *p* < 0.05).

To further investigate the biological function of *LsaPHR1.1*, we knocked out the *LsaPHR1.1* using the CRISPR/cas9 system. Three homozygous mutants with low *LsaPHR1.1* expression (Figure S1) were selected for further analysis under phosphorus stress. Specifically, the KO29-1 allele had five nucleotide deletions, and the KO29-2 and KO30-7 alleles had 1 and 2 bp insertions, respectively (Figure 6A). Then, the main agronomic traits (phosphorus content, plant height, number of leaves, and leaf length and width) were examined.

There were no significant differences between the mutants and the wild-type in these traits (Figure 6C–H; Tables S1–S6). The results indicate that the *LsaPHR1.1* mutation had little effect on normal plant growth and phosphorus uptake.

### 2.7. Lsaphr1.1 Improves Nitrogen Level, Antioxidant Capacity, and Photosynthesis, Improving Phosphorus Stress Tolerance in Lettuce

**Figure 7:**
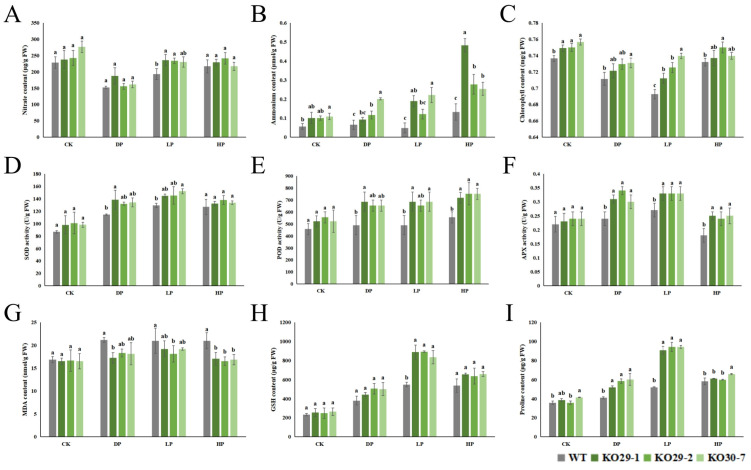
Physiological parameters measured in wild-type and transgenic lettuce subjected to phosphorus stress. (**A**) Nitrate levels. (**B**) Ammonium levels. (**C**) Chlorophyll content. (**D**) Superoxide dismutase (SOD) activity. (**E**) Peroxidase (POD) activity. (**F**) Ascorbate peroxidase (APX) activity. (**G**) Malondialdehyde (MDA) content. (**H**) Glutathione (GSH) levels. (**I**) Proline content. Vertical bars represent mean ± SE of triplicate measurements, and different superscript letters show significant variation (0.01 < *p* < 0.05).

Plants typically adjust their nutrient preference, shifting from one nutrient to others when the primary source becomes limited in the environment [15]. As shown in Figure 7A,B, the *LsaPHR1.1* mutants improved the nitrate and ammonium contents of lettuce to a certain degree. Furthermore, compared to WT plants, chlorophyll content of transgenic lines was increased (Figure 7C). At the same time, the activities of key antioxidant enzymes—including superoxide dismutase (SOD), peroxidase (POD), and ascorbate peroxidase (APX)—were increased in the transgenic lines compared to WT (Figure 7D–F). As key indicators of oxidative damage, the contents of malondialdehyde (MDA), glutathione (GSH), and proline were also measured (Figure 7G–I). Transgenic plants showed increased accumulation of both GSH and proline, along with decreased MDA content relative to wild-type controls, demonstrating improved oxidative stress tolerance and membrane stability. These findings demonstrate that the *LsaPHR1.1* mutants enhance phosphorus stress tolerance in lettuce by enhancing antioxidant enzyme activity and maintaining photosynthetic efficiency.

## 3. Discussion

As a fundamental macronutrient, phosphorus (P) is crucial for multiple aspects of plant physiology and productivity [16]. Global agricultural systems face significant challenges due to poor phosphorus use efficiency in cultivated soils, which substantially constrains yield potential and exacerbates food supply concerns [17]. Enhancing phosphorus acquisition and assimilation efficiency therefore represents a critical strategy for sustainable crop improvement. Molecular investigations have identified numerous genetic components mediating low-P adaptation, among which the MYB transcription factor PHR has emerged as a central regulator of phosphate starvation responses [18,19,20]. Multiple plant species have been found to possess characterized *PHR1* genes. In this study, a total of five *PHR1* genes were identified in the lettuce genome, which is equivalent in number to tobacco (4). Comparing their functional domains and gene structures showed that all LsaPHR1s have Myb_CC_LHEQLE domains and Myb_SHAQKYF domains, which is similar to multiple species’ PHR1s [5,21,22,23]. The similar intron–exon organization of *LsaPHR1.1* through *LsaPHR1.5* suggests their close evolutionary relationship. The results of chromosomal localization analysis have shown that both *LsaPHR1.1* and *LsaPHR1.3* are located on chromosome 9, indicating that they may be homologous genes arising from fragment replication.

Cis-regulatory elements serve as critical mediators of transcriptional control, enabling plants to adapt their gene expression patterns to environmental cues and developmental needs [24]. Research has demonstrated that phosphate deficiency responses are coordinately controlled by multiple signaling pathways, including photoreception, carbohydrate metabolism, and phytohormone signaling (auxin, ethylene, cytokinin, and gibberellin), in addition to oxygen availability [25,26,27,28]. A representative example is Arabidopsis *AtPHR1*, whose expression shows dual regulation by both light and ethylene signals, with these responses being integrated through specific cis elements in the *AtPHR* promoter region [29]. Our analysis revealed multiple cis-acting elements within the promoter regions of *LsaPHR1* genes, and a large number of light-responsive and hormone-related elements indicate that the expression and regulation of *LsaPHR1* genes are influenced by light and hormones. Therefore, the expression patterns of *LsaPHR1*s under various stresses, including DP, LP, HP, darkness, ABA, IAA, and MeJA treatments, were analyzed. The results indicate that the expressions of *LsaPHR1*s were regulated by the aforesaid stresses, suggesting their involvement in phosphorus stress, hormone, and darkness response pathways in lettuce. The expression patterns of *LsaPHR1*s under different treatments are complex, indicating that there is no correlation between the expression levels of these genes and the duration of experimental treatment. For example, during DP treatment, *LsaPHR1.1* and *LsaPHR1.5* exhibited rapid upregulation, followed by rapid downregulation at 6 h and an increase by 12 or 24 h. However, *LsaPHR1.2* and *LsaPHR1.3* exhibited rapid downregulation, followed by upregulation at 12 h. In addition, *LsaPHR1* genes with closer evolutionary relationships show similar expression patterns, indicating their similar functions. Certain genes show irregular patterns of expressions, implying that their expressions might be regulated by other environmental factors (light, temperature, etc.).

To investigate the biological function of *LsaPHR1.1*, gene editing technology was used to generate *LsaPHR1.1* lettuce mutants and subject them to phosphorus stress. The results indicate that editing *LsaPHR1.1* has little effect on lettuce morphology and phosphorus content. As a fundamental macronutrient, nitrogen (N) plays vital roles in various physiological processes throughout plant development. Vegetative organisms possess the capacity to assimilate diverse nitrogenous compounds from their rhizosphere environment, including inorganic ions (nitrate and ammonium) as well as organic nitrogen sources (glutamic acid, glutamine, and urea) [30,31,32]. Research has demonstrated significant crosstalk between nitrogen and phosphorus signaling networks in plants. Elevated nitrate concentrations have been shown to promote phosphate (Pi) absorption, while Pi-deprived conditions inhibit nitrate assimilation while simultaneously enhancing ammonium uptake [33,34]. Under Pi limitation, plants exhibit increased ammonium absorption and accumulation of the STOP1 transcription factor. This protein triggers organic acid secretion, which facilitates the release of bound phosphorus from insoluble iron/calcium phosphate complexes, ultimately improving Pi acquisition [35]. A recent report revealed that nitrate serves as a powerful regulator in modulating the activation of the SPX1 promoter, which is induced by phosphorus (Pi) starvation [36]. In this study, editing *LsaPHR1.1* improved the nitrate and ammonium contents of lettuce to a certain degree. Hence, it can be deduced that the mutants maintained phosphorus and growth levels by coordinating N and P signaling pathways. Further research is required.

As crucial signaling intermediaries, reactive oxygen species (ROS) play pivotal roles in mediating plant stress adaptation and developmental processes [37]. The regulation of ROS in plants is mediated by an intricate antioxidant network, which incorporates enzymatic antioxidants (e.g., superoxide dismutase, peroxidase, ascorbate peroxidase) alongside non-enzymatic antioxidants like glutathione and proline [38]. Phosphorus deficiency has been shown to elevate ROS generation while simultaneously activating antioxidant mechanisms [39,40]. In transgenic plants under Pi stress, enhanced activities of SOD, POD, and APX enzymes were observed, facilitating ROS scavenging. These plants exhibited decreased malondialdehyde accumulation, suggesting reduced membrane damage from oxidative stress. Elevated glutathione and proline content further contributed to cellular redox balance. Notably, transgenic lines maintained superior chlorophyll retention compared to wild-type counterparts, demonstrating improved photosynthetic performance under phosphorus limitation. The direct molecular mechanisms (upstream and downstream targets of *LsaPHR1.1*) require further study.

All in all, editing *LsaPHR1.1* in lettuce enhances its tolerance to phosphorus stress by mediating the N–P interaction, boosting antioxidant enzyme activity, elevating antioxidant content, and preserving photosynthetic efficiency. In the future, the overexpression of *LsaPHR1* genes in lettuce will be planned for further functional validation, with the aim of enhancing the efficiency of phosphorus utilization in lettuce. Furthermore, the functional redundancy or divergence among the five *LsaPHR1* genes will be fully explored.

## 4. Methods and Materials

### 4.1. Plant Materials

From March to April 2025, experimental trials were performed in a solar greenhouse situated at the Vegetable Research Institute under the Beijing Academy of Agricultural and Forestry Sciences (coordinates: 116°29′ E, 39°94′ N). Lettuce cultivar LVYA was used as the experimental material. Lettuce seeds were sown in a growing medium (peat/vermiculite/perlite = 2:1:1). The seedlings were treated with 1/4 Hoagland nutrient solution (Table S7). Following 14 d of growth, the plants were exposed to various stress treatments: phosphorus treatments consisted of 0 μmol·L^−1^ phosphate for devoid phosphorus stress (DP), 80 μmol·L^−1^ phosphate for low phosphorus stress (LP), and 1000 μmol·L^−1^ phosphate for high phosphorus stress (HP). Leaf samples were collected at 0, 2, 6, 12, and 24 h after spraying on the leaves. Untreated leaves collected prior to spraying were designated as the 0-h control group. For the dark treatment, the plants were exposed to a 0-h light period (sampling at 0, 2, 6, 12, and 24 h). For phytohormone treatments, the leaves were sprayed with 100 µmol L^−1^ abscisic acid (ABA), 100 µmol L^−1^ auxin (IAA), and 10 µmol L^−1^ methyl jasmonate (MeJA). Leaf tissues were harvested at five intervals (0 h, 2 h, 6 h, 12 h, and 24 h). For each treatment group, leaves from randomly chosen plants were gathered, with three biological replicates included (each containing three plants). Upon collection, all samples were rapidly frozen using liquid nitrogen and maintained at −80 °C until further processing.

### 4.2. Identification and Characterization of the LsaPHR1 Gene Family

To identify homologous genes, the PHR1 protein sequence was obtained from the *Arabidopsis thaliana* database (http://Arabidopsis.org, accessed on 6 March 2025) and aligned against the lettuce genome using NCBI’s BlastP function (http://blast.ncbi.nlm.nih.gov, accessed on 6 March 2025), applying stringent criteria (E-value cutoff: 1.0 × 10^−10^; minimum identity: 40%). Next, to exclude non-conforming sequences, the candidate genes were analyzed for conserved domains using the online CDD platform (http://ncbi.nlm.nih.gov/cdd, accessed on 7 March 2025) [41]. Additional protein characteristics including amino acid composition, molecular mass, theoretical pI, stability index, and hydropathicity profile were determined through ExPASy’s ProtParam tool (http://web.expasy.org/protparam/, accessed on 7 March 2025) [42]. Subcellular localization predictions were performed using Cell-PLoc 2.0 (http://www.csbio.sjtu.edu.cn/bioinf/Cell-PLoc-2/, accessed on 7 March 2025) [43].

### 4.3. Multiple Sequence Alignment and Phylogenetic Tree Construction

The protein sequences of tobacco PHR1 family members were retrieved from the NCBI database (https://www.ncbi.nlm.nih.gov/, accessed on 7 March 2025). Phylogenetic analysis and multiple sequence alignment were conducted using MEGA 11, comparing PHR1 homologs from Arabidopsis, tobacco, and lettuce. The phylogenetic reconstruction employed 1000 bootstrap iterations to assess nodal support.

### 4.4. Conservation and Structural Analysis of LsaPHR1

The conserved protein motifs of LsaPHR1 family members were identified using the MEME suite (http://meme-suite.org/, accessed on 8 March 2025), with parameter settings configured to detect five distinct motifs [44]. Additionally, gene structure analyses including exon–intron organization were performed through the GSDS 2.0 online platform (http://gsds.cbi.pku.edu.cn, accessed on 8 March 2025) [45].

### 4.5. Chromosomal Location and Cis-Acting Element Analysis

Chromosomal localization of *PHR1* genes was mapped using lettuce genome data, followed by construction of genetic linkage maps with TBtools-II (ver. 2.1) software [46]. For cis element analysis, we extracted 2000 bp promoter sequences upstream of each gene’s initiation codon. These promoter regions were then examined using PlantCARE (http://bioinformatics.psb.ugent.be/webtools/plantcare/html/, accessed on 9 March 2025) to identify regulatory elements [47]. Data visualization was performed using Microsoft EXCEL 2016.

### 4.6. Expression Analysis by qRT-PCR

The expression patterns of *LsaPHR1*s under diverse abiotic stress treatments (DP, LP, HP, darkness, ABA, IAA, and MeJA) were examined using quantitative real-time PCR (Bio-Rad, Hercules, CA, USA). RNA extraction was performed on plant samples using the FastPure Universal Plant Total RNA Isolation Kit (Vazyme, Nanjing, China). For cDNA synthesis, 1 µg of purified total RNA was processed with the HiScript III 1st Strand cDNA Synthesis Kit (+gDNA wiper) from the same manufacturer. Quantitative reverse transcription PCR (qRT-PCR) was conducted on a Bio-Rad CFX Opus 96 instrument (Hercules, CA, USA) to determine cycle threshold (C_t_) values. The reaction mixture (20 µL total volume) contained 10 µL of 2× SYBR Green Master Mix (TOYOBO, Osaka, Japan), 0.5 µL each of forward and reverse primers, and 2 µL cDNA template, with the remaining volume consisting of nuclease-free water. The experimental design incorporated triplicate biological replicates, using *18S rRNA* as an internal control. The 2^−∆∆Ct^ method was used to calculate the relative gene expression levels [48] (Table S8).

### 4.7. The Genetic Transformation of LsaPHR1.1 and the Experimental Treatments of Their Offsprings

gRNA was designed using the CRISPOR web tool, prioritizing gene specificity to avoid off-target editing [49]. The gRNA was cloned into the pZKD672 vector. The constructs were transformed into wild-type (WT) lettuce (LVYA) using the agrobacterium-mediated cotyledon infection method. A detailed procedure can be found in a previous publication [50]. Then, generating gene-edited lines for *LsaPHR1.1,* the transformants and their offspring were screened.

T3 homozygous lines (KO29-1, KO29-2, and KO30-7) and WT were selected for subsequent phenotypic analysis and phosphorus stress assays. Specifically, when the lettuce sprouted seven true leaves, the plants were subjected to various phosphate concentrations: 1000 μmol·L^−1^ phosphate for the control group (CK), 0 μmol·L^−1^ phosphate for devoid phosphorus stress (DP), 200 μmol·L^−1^ phosphate for low phosphorus stress (LP), and 2000 μmol·L^−1^ phosphate for high phosphorus stress (HP). After 9 d of hydroponic cultivation (grown in Hoagland solution modified to contain the aforementioned phosphate concentrations), the phenotypes were analyzed and the physiological indices were determined.

### 4.8. Phenotypic Analysis and Determination of Physiological Indices

Morphological parameters, including plant height and leaf dimensions (length and width), were recorded using standard measuring rulers. Chlorophyll quantification was performed through ethanol-based extraction followed by colorimetric analysis [51]. Enzyme activities (SOD, POD, and APX) and biochemical markers (MDA, proline, and GSH) were assayed using commercial kits (Solarbio, Beijing, China) following the manufacturer’s instructions. Nutrient analysis (nitrate, ammonium, and total phosphorus) was conducted with the same kit series. *LsaPHR1.1* expression was quantified as previously described. All measurements were performed in triplicate biological replicates.

### 4.9. Statistical Analysis

Statistical analysis was performed using PASW Statistics 18 (employing both LSD and Waller–Duncan tests) to determine significant differences among treatment groups. Experimental data processing and graphical representation were accomplished with EXCEL 2016. In the figures below, treatments marked with distinct letters show statistically significant variation (0.01 < *p* < 0.05).

## 5. Conclusions

This study employed bioinformatics methods to systematically identify and analyze five *PHR1* genes in lettuce. The expression profiles of *LsaPHR1*s were further investigated under various conditions, including phosphorus deficiency, low and high phosphorus levels, darkness, and treatments with ABA, IAA, and MeJA, using quantitative real-time PCR (qRT-PCR). Additionally, we generated transgenic lettuce by editing *LsaPHR1.1* to validate its function in enhancing phosphorus (DP, LP, and HP) tolerance in lettuce. The results indicate that *LsaPHR1*s in lettuce are involved in pathways related to responses to phosphorus stress, hormones, and darkness. Editing *LsaPHR1.1* enhances the tolerance of lettuce to phosphorus stress by mediating the N–P interaction, increasing antioxidant enzyme activity, elevating antioxidant content, and maintaining photosynthetic efficiency. This research presents a potential strategy for enhancing phosphorus stress tolerance in lettuce, which is crucial for maintaining both yield and quality.

## Figures and Tables

**Table 1:** PHR1 proteins in lettuce.

Gene Name	Gene ID	Molecular Weight (Da)	No. of Amino Acids	pI	Instability Index	Grand Average of Hydropathicity	Subcellular Localization
*LsaPHR1.1*	LOC111881312	48,138.67	435	5.55	66.66	−0.789	Nucleus
*LsaPHR1.2*	LOC111895520	45,858.76	418	5.51	61.23	−0.759	Nucleus
*LsaPHR1.3*	LOC111903878	45,775.19	410	5.30	47.47	−0.786	Nucleus
*LsaPHR1.4*	LOC111895914	30,769.89	281	6.10	53.52	−0.41	Nucleus
*LsaPHR1.5*	LOC111915887	28,849.99	261	6.60	52.03	−0.458	Nucleus

## Data Availability

The data presented in this study are openly available in NCBI at https://www.ncbi.nlm.nih.gov/.

## Supplementary Materials

The following supporting information can be downloaded at: https://www.mdpi.com/article/10.3390/ijms262110466/s1.
