# Supplementary material for: Genome-Wide Identification of the LsaPHR1 Gene Family and Preliminary Functional Validation of LsaPHR1.1 in Phosphorus Tolerance in Lactuca sativa
Source: Int J Mol Sci. 2025 Oct 28;26(21):10466. doi: 10.3390/ijms262110466 (PMC12609090; doi:10.3390/ijms262110466)
Supplement: Supplementary file 1 [file ijms-26-10466-s001.zip › ijms-3825692-supplementary.pdf]

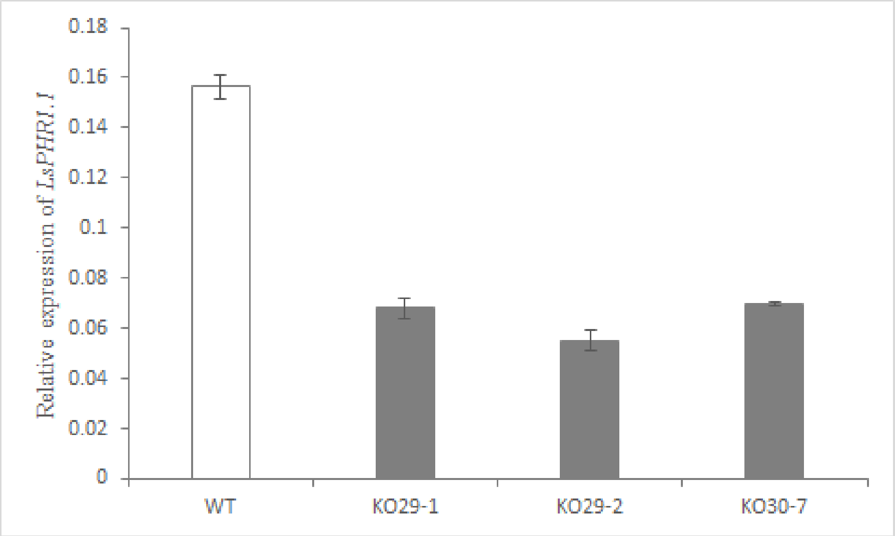

Figure S1. Knockout results of qRT-PCR verifcation.

Table S1. Leaf number.

	CK	DP	LP	HP
WT	6.67 ± 0.47	6.67 ± 0.47	6.00 ± 0.82	6.67 ± 0.47
KO29-1	7.33 ± 0.47	7.33 ± 0.47	7.00 ± 0.82	6.00 ± 0.82
KO29-2	7.00 ± 0.82	7.33 ± 0.47	7.00 ± 0.82	6.00 ± 0.82
KO30-7	7.33 ± 0.47	7.67 ± 0.47	7.33 ± 0.47	6.33 ± 0.47

Table S2. Plant height.

	CK	DP	LP	HP
WT	15.47 ± 0.69	14.30 ± 0.54	15.43 ± 0.87	13.43 ± 0.05
KO29-1	15.33 ± 0.99	14.20 ± 0.37	15.80 ± 0.85	13.73 ± 0.29
KO29-2	15.17 ± 0.37	14.27 ± 0.31	15.67 ± 0.26	13.73 ± 0.66
KO30-7	15.57 ± 1.02	14.43 ± 0.34	15.77 ± 0.45	13.67 ± 1.10

Table S3. Leaf length.

	CK	DP	LP	HP
WT	14.60 ± 0.37	13.97 ± 0.62	14.47 ± 0.54	13.07 ± 0.09
KO29-1	14.83 ± 0.50	13.93 ± 0.37	15.10 ± 0.49	13.43 ± 0.94
KO29-2	14.70 ± 0.29	13.70 ± 0.36	15.07 ± 0.86	13.17 ± 0.86
KO30-7	14.73 ± 0.82	13.90 ± 0.24	15.43 ± 0.29	13.20 ± 0.57

Table S4. Leaf width.

	CK	DP	LP	HP
WT	7.27 ± 0.12	6.73 ± 0.26	6.67 ± 0.48	6.70 ± 0.29
KO29-1	7.57 ± 0.29	6.90 ± 0.65	6.93 ± 0.29	6.33 ± 0.53
KO29-2	7.50 ± 0.42	6.80 ± 0.45	7.00 ± 0.29	6.10 ± 0.37
KO30-7	7.80 ± 0.22	6.93 ± 0.69	6.97 ± 0.25	6.40 ± 0.16

Table S5. Root phosphorus content.

	CK	DP	LP	HP
WT	3.76 ± 0.53	2.06 ± 0.50	2.20 ± 0.10	3.97 ± 0.44
KO29-1	3.83 ± 0.35	2.06 ± 0.20	2.77 ± 0.35	3.97 ± 0.44
KO29-2	3.62 ± 0.35	1.99 ± 0.36	3.19 ± 0.35	4.33 ± 0.27
KO30-7	3.69 ± 0.36	2.27 ± 0.56	2.55 ± 0.35	4.40 ± 0.36

Table S6. Leaf phosphorus content.

	CK	DP	LP	HP
WT	3.62 ± 0.30	1.70 ± 0.63	4.18 ± 0.44	6.60 ± 0.46
KO29-1	3.97 ± 0.66	1.84 ± 0.10	3.40 ± 0.17	6.67 ± 0.50
KO29-2	4.04 ± 0.87	1.42 ± 0.10	3.69 ± 0.44	6.60 ± 0.35
KO30-7	3.90 ± 0.27	1.77 ± 0.10	3.83 ± 0.35	6.88 ± 0.44

Table S7. The components of Hoagland nutrient solution.

Nutrient solution composition	Concentration (mg/L)
$Ca(NO_3)_2$	945
K_2SO_4	607
$NH_4H_2PO_4$	115
$MgSO_4$	493
EDTA ferric-sodium salt	20
$FeSO_4$	15
H_3BO_3	2.86
$Na_2B_4O_7 \cdot 10H_2O$	4.5
$MnSO_4$	2.13
$CuSO_4$	0.05
$ZnSO_4$	0.22
$(NH_4)_2SO_4$	0.02

Note: Hoagland nutrient solution was purchased from Qingdao Hope Bio-Technology Co. , Ltd.

Table S8. The qRT-PCR primers used in this study.

Gene name	Primer sequence (5'-3')
18S RNA	F: GTGAGTGAAGAAGGGCAATG
	R: CACTTTCAACCCGATTCACC
LsaPHR1.1	F: GGCTACACGAACAACTGGAGA
	R: GGGTGTGGGTGGATGATGAA
LsaPHR1.2	F: GAAACAATGCAAGCCAGGAGG
	R: GTCATCATCTGGTCCCGGTC
LsaPHR1.3	F: AGATTGGCAGAACTGGGCTG
	R: GGCACTTGAGGCCTAGGAA
LsaPHR1.4	F: GCCGTTACTCAACTTGGTGG
	R: CAAGATTGTTTCCCCAGCCG
LsaPHR1.5	F: CATCGACTCCAGCTTCGGAT
	R: GCCAGCTCAGATAACTCCTCC
